# Supplementary material for: In Vitro One-Pot 3-Hydroxypropanal Production from Cheap C1 and C2 Compounds
Source: Int J Mol Sci. 2022 Apr 3;23(7):3990. doi: 10.3390/ijms23073990 (PMC8999356; doi:10.3390/ijms23073990)
Supplement: Supplementary file 1 [file ijms-23-03990-s001.zip › ijms-1671068-supplementary.pdf]

Supplementary data

In vitro one-pot 3-hydroxypropanal production from cheap C1 and C2 compounds

Su-bin Ju[1,2†], Min-Ju Seo[2†], and Soo-Jin Yeom[1,2*]

[1] School of Biological Sciences and Biotechnology, Graduate School, Chonnam National University, Yong-bong-ro 77, Gwangju 61186, Republic of Korea

[2] School of Biological Sciences and Technology, Chonnam National University, Gwangju 61186, Republic of Korea

[†] These authors contributed equally to this work

* Correspondence: Soo-Jin Yeom soojin285@chonnam.ac.kr

Table S1. Strain, plasmids, and primers used in this study

Name	Relevant genotype or description	Source
Bacterial strain		
E. coli C2566	*fhuA2 lacZ::T7 gene1 [lon] ompT gal sulA11 R(mcr-73::miniTn10*--Tet^S*)2 [dcm] R(zgb-210::Tn10*--Tet^S*) endA1 Δ(mcrC-mrr)114::IS10*	Enzynomics
Plasmid		
pET-28a(+)	*E. coli* expression vector, Kan^R, T7 promoter	Lab stock
pMDH$_{Lx}$K46E	pET-28a(+) containing MDH$_{Lx}$ K46E variant	Lab stock
pMDH$_{Lx}$A164F	pET-28a(+) containing MDH$_{Lx}$ A164F variant	Lab stock
pMDH$_{Lx}$K318N	pET-28a(+) containing MDH$_{Lx}$ K318N variant	Lab stock
pMDH$_{Lx}$K46E-A164F	pET-28a(+) containing MDH$_{Lx}$ K46E-A164F double variant	This study
pMDH$_{Lx}$A164F-K318N	pET-28a(+) containing MDH$_{Lx}$ A164F-K318N double variant	This study
pDERA$_{Tma}$	pET-28a(+) containing DERA$_{Tma}$	This study
Oligonucleotides used for PCR (5′−3′)		
DERA$_{Tma}$	(F)-gtgccgcgcggcagccatatgatcgaataccgtatcg	This study
	(R)-ggtggtgctcgagttaaccaccgtaacgttc	
Oligonucleotides used for site-directed mutagenesis of MDH$_{Lx}$ (5′−3′)		
A164F	(F)-acagctcgtaaagtgaagatg***ttc***atcgtggacaaacatgttac	This study
	(R)-gtaacatgtttgtccacgat***gaa***catcttcactttacgagctgt	
* Mutagenized codons are highlighted in italic and bold.		

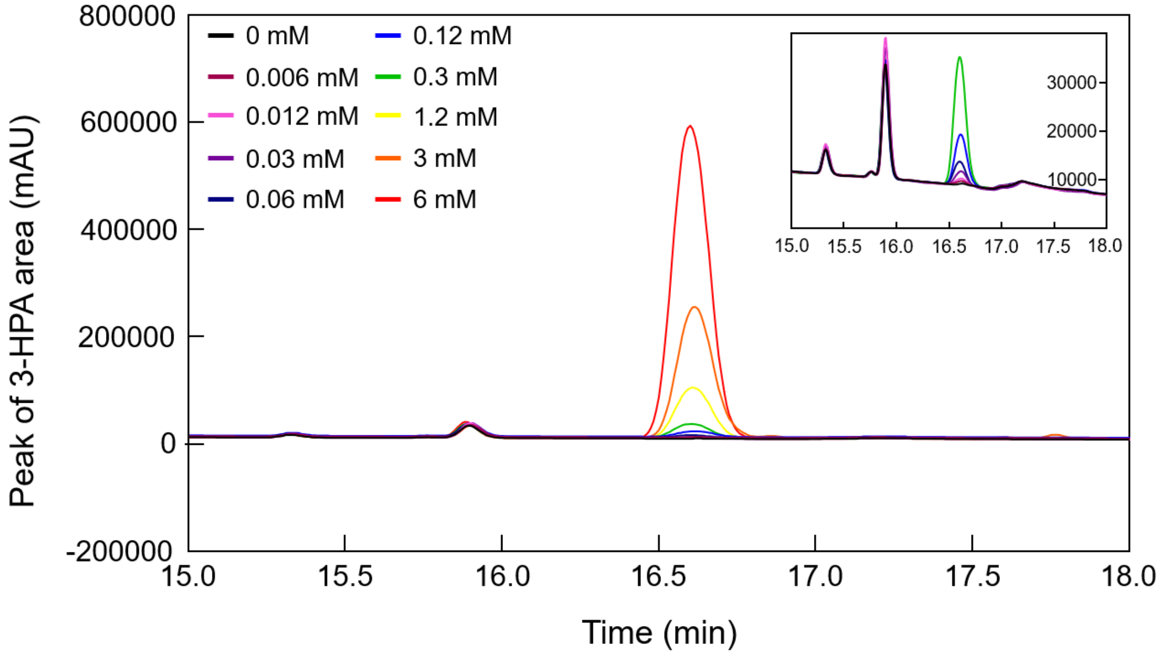

Figure S1. HPLC chromatography of 3-HPA after derivatization with *O*-benzylhydroxylamine.

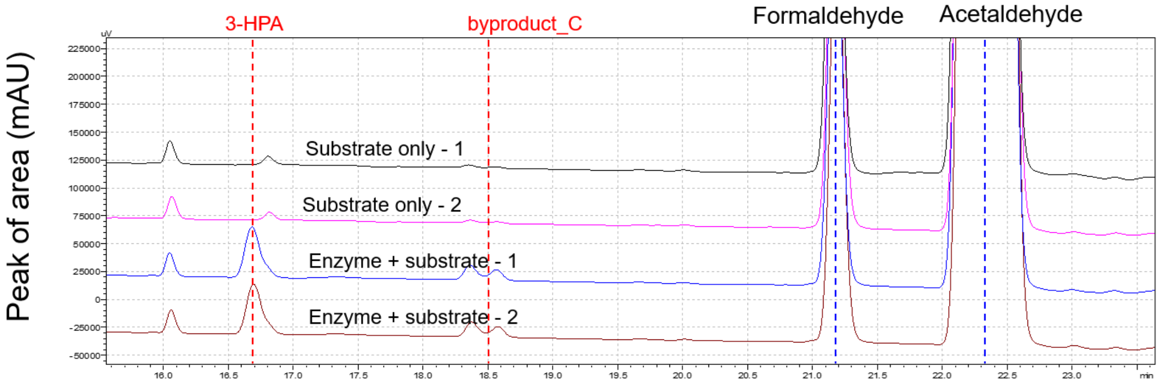

Figure S2. HPLC chromatography of 3-HPA derivatives, formaldehyde, and acetaldehyde.

(a)

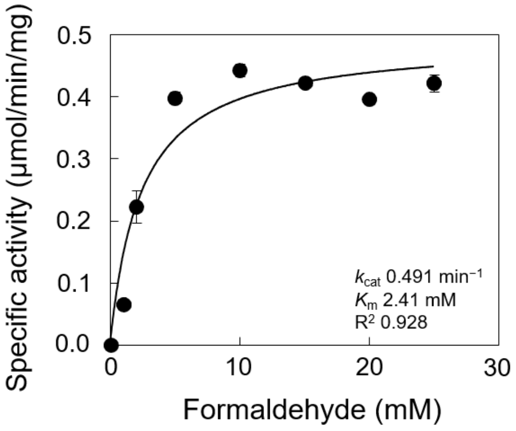

(b)

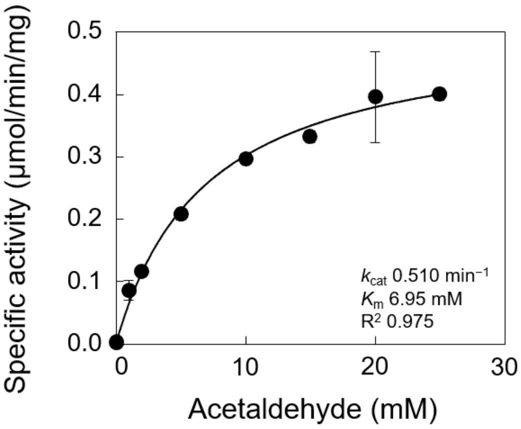

Figure S3. Kinetic parameters of DERA$_{Tma}$. (a) Kinetic parameter of DERA$_{Tma}$ toward formaldehyde. (b) Kinetic parameter of DERA$_{Tma}$ toward acetaldehyde. The reactions were performed in 20 mM Kpi buffer (pH 7.0) containing 0.5 mg/mL enzyme and various concentration of substrates for 10 min at 40 ℃. Data represent the means of two separate experiments, and error bars represent the standard deviation.

(a)

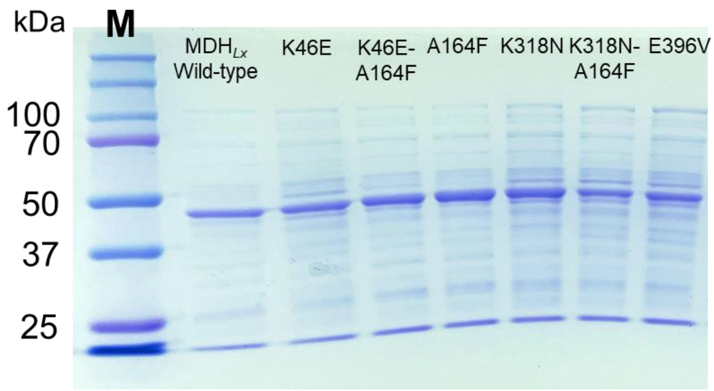

(b)

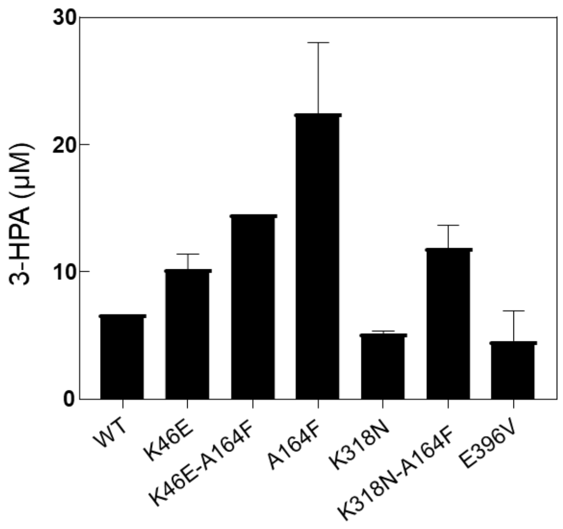

Figure S4. SDS-PAGE analysis and activity test of the wild type MDH$_{Lx}$ and its variants. (a) SDS-PAGE analysis of the purified MDH$_{Lx}$ and its variants (48 kDa). Pre-stained marker protein (250, 150, 100, 70, 50, 37, and 25 kDa) and purified enzymes were loaded. (b) Comparison of enzyme activities with MDH$_{Lx}$ and its variants. The reactions were performed in 20 mM Kpi buffer (pH 7.0) containing 2.22 U/mL DERA$_{Tma}$, 0.01 U/mL MDH$_{Lx}$ and its variants, 1 M methanol, 1M ethanol, 5 mM Mg^{2+}, and 3 mM NAD^{+} at 45 ℃ for 120 min. Data represent the means of two separate experiments, and error bars represent the standard deviation.

A

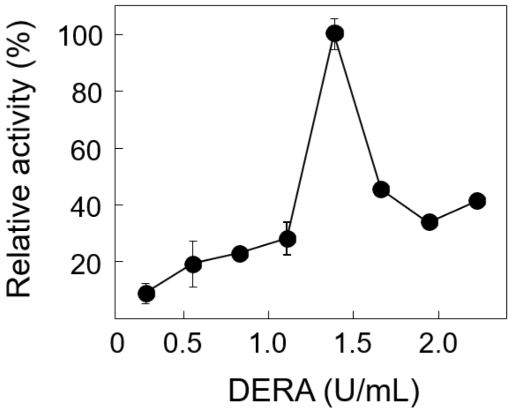

B

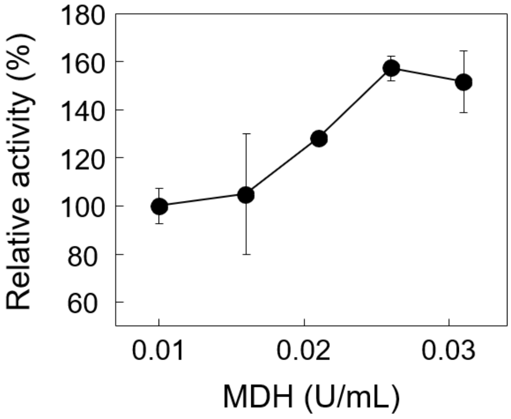

Figure S5. Effect of enzyme concentration for 3-HPA production. (A) Effect of DERA$_{Tma}$ concentration. (B) Effect of MDH$_{Lx}$ A164F concentration. The reactions were conducted in 20 mM Kpi buffer (pH 7.0) containing 1 M methanol, 1M ethanol, 5 mM Mg^{2+}, 3 mM NAD^{+}, and different concentration of enzymes at 45 ℃ for 120 min. Data represent the means of two separate experiments, and error bars represent the standard deviation.
